# Mirror alignment by particle swarm optimization for synchrotron radiation beamlines

**DOI:** 10.1107/S1600577526008994

**Published:** 2026-10-05

**Authors:** Bunyo Okumura, Kunihiro Goto, Akinobu Fujii, Takamasa Nonaka, Tsukasa Shimizu, Yoshihiro Ohama

**Affiliations:** a Toyota Central R&D Laboratories Inc., 41-1 Yokomichi, Nagakute, Aichi480-1192, Japan; Advanced Photon Source, USA

**Keywords:** synchrotron radiation beamline, mirror alignment, particle swarm optimization

## Abstract

This article proposes a particle swarm optimization (PSO)-based automated mirror-alignment methodology that enables the utilization of high-flux beams for measurements in synchrotron radiation beamlines. The method is designed to improve beam flux by adjusting two mirrors within a target alignment time of 5 min, thereby satisfying critical operational demands. The proposed PSO-based method incorporates three adaptations: (i) particle initialization and dynamics (which are modified to locate a narrow flux peak efficiently), (ii) mirror-movement optimization (which reduces the total mirror-travel time), and (iii) a termination condition (which balances the practical trade-off between alignment time and beam flux). Simulation results demonstrate the feasibility of achieving the alignment target and show that under the evaluated conditions the proposed method identified high-flux solutions more consistently than a general-purpose Gaussian-process-based Bayesian optimization method. Further, validation over a 15 month deployment period on an operational beamline, during which the proposed method improved beam flux in 107 of 124 runs (86.3%), demonstrates its robustness and reliability.

## Abstract 2

A particle swarm optimization-based automated two-mirror alignment method was developed to restore high-flux beams in approximately 5 min, and its 15 month deployment demonstrated its robustness and reliability.

## 1. Introduction

Synchrotron radiation enables advanced materials characterization and analysis. Beamline experiments require high-flux X-ray beams to reduce acquisition time and improve the signal-to-noise ratio. A synchrotron beamline comprises multiple optical elements that must align precisely to maximize the delivered X-ray flux. Despite meticulous optimization, the beam flux frequently degrades over time owing to changes in storage-ring operating conditions and thermal drift within the optical elements.

In practice, the manual tuning of optical elements is time-consuming, requiring an automated alignment technique that features the following properties to maximize beam-time utilization:

(i) Increases the beam flux.

(ii) Completes the alignment within a short time.

(iii) Does not degrade the beam flux even if the alignment attempt fails.

Thus far, numerous studies have explored the automated alignment of optical elements, which remains a fundamental challenge for synchrotron beamlines. Such automation is typically achieved by replacing manual alignment with rule-based programs (Mangold, 2018 ▸; Svensson & Pugliese, 1998 ▸; Stepanov *et al.*, 2022 ▸; Zhou *et al.*, 2019 ▸; McPhillips *et al.*, 2002 ▸; Gabadinho *et al.*, 2010 ▸). Model-based closed-loop optimization methods have been proposed to optimize mirror positions and to enable real-time alignment (de La Rochefoucauld *et al.*, 2021 ▸; Zhang *et al.*, 2023*a* ▸).

Conversely, a synchrotron beamline comprises multiple optical elements, resulting in a high-dimensional parameter space with more than ten adjustment parameters and a nonlinear objective response to these inputs.

Consequently, several methodologies rely on black box optimization frameworks as the adjustment algorithm. Black box optimization comprises a class of methodologies that seek optimal solutions exclusively via objective function evaluations for specific inputs. These methodologies are particularly suitable when a mathematical model of a system is unavailable or when constructing a high-fidelity machine learning (ML) model is expensive.

Evolutionary algorithms (EAs) account for one category of black box optimization methodologies (Hertz & Kobler, 2000 ▸). EA is an umbrella term for approaches that treat a set of parameters as an individual and refine candidate solutions via iterative mutation and selection within a population of parameter sets. A foremost methodology in EAs is the genetic algorithm (GA) (Holland, 1975 ▸), which represents parameters as discretized, encoded genes and performs the search via mutation and crossover operations. Additionally, differential evolution (DE) (Storn & Price, 1997 ▸) has been proposed to represent parameters in a continuous search space as real-valued vectors.

Numerous GA-based methodologies have been proposed for the alignment of optical elements (Xi *et al.*, 2015 ▸; Xi *et al.*, 2017 ▸; Zhang *et al.*, 2023*b* ▸; Zhang *et al.*, 2023*c* ▸). For instance, GA was implemented at the X-ray absorption fine-structure/chemical analysis (XAFCA) beamline of the Singapore Synchrotron Light Source (SSLS) to enhance the sample-position photon flux. This was achieved by adjusting 14 parameters across three optical elements (Xi *et al.*, 2015 ▸). Further, a LabVIEW-based program integrating GA and DE was developed to provide graphical user interface (GUI)-based functions, such as algorithm initialization and termination, for the XAFCA and 1W1B beamlines of the SSLS and Beijing Synchrotron Radiation Facility (BSRF), respectively (Xi *et al.*, 2017 ▸). The study achieved alignment times of approximately 10 and 15 min for DE and GA, respectively. A GA-based multi-objective optimization method was proposed for the ID17 beamline at the European Synchrotron Radiation Facility (ESRF) to simultaneously maximize beam flux and energy (Zhang *et al.*, 2023*b* ▸). By adjusting parameters, such as the gas-filter length and position, the solid-filter position, and the ionization-chamber position, they realized Pareto fronts that reflect the trade-off between beam flux and energy. A specialized system featuring a GUI was developed for flux maximization and spot-position adjustment at the BL13U hard X-ray nanoprobe beamline of the Shanghai Synchrotron Radiation Facility (SSRF) (Liu *et al.*, 2024 ▸). The system employed the non-dominated sorting genetic algorithm II (NSGA-II), a widely utilized GA variant. By storing previous alignment results in a database and reusing them for optimization, the system reduced the alignment time, requiring approximately 2 and 9 min for single- and double-objective four-axis alignments, respectively.

Other black box optimization methods include particle swarm optimization, PSO (Kennedy & Eberhart, 1995 ▸), and Bayesian optimization, BO. PSO is a heuristic optimization algorithm that is formulated by modeling collective social behaviors observed in biological swarms, such as birds and fish. PSO, the artificial Bee Colony, NSGA-II, and GA have been compared via simulations using an X-ray tracer (Karaca *et al.*, 2024 ▸). The study revealed that PSO achieved the best performance in single-objective optimization for spot-size minimization or flux maximization. The authors also reported that multi-objective optimization for spot-size minimization and flux maximization highlighted a trade-off along the NSGA-II-generated Pareto front. BO represents an effective methodology for problems involving expensive sampling. An extant study (Morris *et al.*, 2024 ▸) proposed a BO-based alignment method and demonstrated the Blop framework that integrates BO with a kernel that learns latent, coupled beamline dimensions. The framework also incorporates probabilistic constraints that account for diagnostic failures and invalid regions, thereby addressing beamline-specific problems. As key demonstrations, the authors explored the six-axis alignment of Kirkpatrick–Baez (KB) mirrors for flux-density maximization at a tender X-ray microspectroscopy beamline (TES 8-BM) of the National Synchrotron Light Source II (NSLS-II). They also attempted the five-axis alignment of a Johann spectrometer for the same objective at the Inner Shell Spectroscopy beamline (ISS 8-ID) of the same NSLS-II facility. To realize high resolution, multi-objective BO was implemented for the alignment of bendable KB mirrors, thus extending the methodology (Rebuffi *et al.*, 2023 ▸). By integrating real-time wavefront metrology with digital twin-based transfer learning, this approach reduced alignment times from several hours to within 5 min. Domain-knowledge-guided BO has been proposed for complex optical alignment by incorporating physics-derived coordinate transformations to decouple coupled control parameters and align the active subspaces with the search axes, together with a scheduled exploration strategy (Mishra *et al.*, 2026 ▸). These studies demonstrate that the performance of BO in beamline alignment can be substantially improved by incorporating problem-specific representations, constraints, prior knowledge, and failure-handling strategies.

Recently, methodologies relying on simulation and experimental data were proposed for the construction of predictive ML models of alignment targets and for performing alignments based on the constructed models (Morris *et al.*, 2022 ▸; Mishra *et al.*, 2023 ▸; Yan *et al.*, 2022 ▸; Xie *et al.*, 2023 ▸; Rebuffi *et al.*, 2025 ▸; Feuer-Forson *et al.*, 2026 ▸). In addition, methods have been proposed that learn alignment policies by reinforcement learning (Bruchon *et al.*, 2020 ▸).

Although various adjustment targets and methodologies have been proposed, only a few studies have addressed the required adjustment time (Xi *et al.*, 2017 ▸; Liu *et al.*, 2024 ▸; Feuer-Forson *et al.*, 2026 ▸; Rebuffi *et al.*, 2023 ▸). This knowledge gap likely stems from the lack of a need for frequent adjustments in many applications and the complexity involved with estimating on-site adjustment time via simulation-based evaluations.

In this study, we propose an optical alignment method and tool that is deployable on an operational beamline and demonstrate its practical performance. We consider the SPring-8 Toyota beamline, BL33XU (Nonaka *et al.*, 2016 ▸), which supports diverse measurement modalities, including X-ray absorption spectroscopy, X-ray computed tomography/computed laminography, and X-ray diffraction. In this manual workflow, operators alternate between moving the mirrors and performing slit scans to acquire beam profiles. After each slit scan, the operators determined the next mirror displacement based on beam profiles, repeating this cycle until the beam flux is restored. This procedure is time-consuming and places a substantial burden on operators. Moreover, since the displacement direction and magnitude are selected via trial and error, the search may fail to explore a sufficiently broad region. To maximize beam-time utilization, available time should be devoted to measurements rather than optical alignment.

To address these limitations, we develop an alignment tool to control a mirror subset (Section 2) and increase the beam flux in less than 5 min. The contributions of this study are highlighted below:

(i) A PSO-based alignment algorithm is proposed to reduce the total alignment time, including actuator motion, measurement, and algorithm computation.

(ii) Simulations are performed to demonstrate the effectiveness of the proposed algorithm.

(iii) The simulation results are validated through continuous operation on an actual beamline.

The remainder of this paper is organized as follows: Section 2 describes the configuration of the target beamline and formulates the optical-element tuning problem. Section 3 presents an optimized PSO-based algorithm that enables rapid, robust tuning. Section 4 demonstrates the effectiveness of the proposed optimization method via simulations, including an ablation analysis and a comparison with a general-purpose Gaussian-process BO (GP–BO) implementation. Section 5 reports the validation results obtained from continuous operation on an actual beamline. Section 6 summarizes the main findings of the study.

## 2. Problem definition

Fig. 1 ▸ illustrates the optical layout of BL33XU for XAFS measurements. X-rays generated by the undulator are first deflected by mirrors 1 (M1) and 2 (M2), after which they are monochromated by channel-cut crystal monochromators 1 (CMono1) or 2 (CMono2), depending on the experimental configuration. Thereafter, the beam axis is deflected by mirrors 3 (M3) and 4 (M4), after which the beam is delivered to the sample.

The position and direction of the synchrotron radiation beam depend on the operating conditions of the storage ring. To maintain high flux under these variations, the M1 and M2 mirrors must first be readjusted. Although each of the M1 and M2 mirrors has five degrees of freedom—translations along the *X* and *Z* axes, tilts about the *X* and *Z* axes, and bending—readjustment of all axes requires a substantial amount of time. Notably, M1 and M2 are 1 m-long mirrors; therefore, adjustments along each axis must be performed at low speed due to their large size. Consequently, each alignment iteration incurs a substantial motion overhead, which increases the total time required for mirror alignment.

In this study, the adjustment targets are limited to the *X*-axis actuators of M1 and M2. The complete adjustments of M1 and M2 facilitate the automatic alignment of the downstream mirrors and slits. Their alignment can be executed via automated algorithms.

The objective is to maximize the flux measured by an ionization chamber installed at the sample position. Let the optical-element parameter be **x** ∈ *R*^2^, and let the synchrotron X-ray flux measured by the counter be *y* ∈ *R*.

The corresponding relationship is modeled as follows: 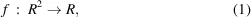

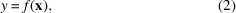

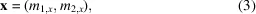
where *m*_1,*x*_ and *m*_2,*x*_ denote the *X*-axis positions of M1 and M2, respectively. Thus, the mirror-alignment problem is formulated as searching for **x** that maximizes the objective function *f*(·).

## 3. Mirror-alignment algorithm

This study primarily aims to minimize the time required for mirror alignment, while maximizing the beam flux. Since the alignment depends on only two mirror-position parameters, the flux response within the operational range may exhibit a single dominant peak corresponding to the optimal optical alignment. Within this setting, an optimization strategy that maintains a shared estimate of the best solution and directs the search toward the global optimum is particularly suitable. PSO provides this capability by integrating global-best guidance with robustness against noisy flux measurements. Consequently, this study adopts PSO as the mirror-alignment methodology.

### 3.1. Particle swarm optimization

PSO is a heuristic algorithm in which a set of randomly initialized search points, called particles, forms a swarm. Each particle explores the solution space by updating its velocity based on its search history and information shared within the swarm. The core mechanism of PSO includes integrating particle-specific and swarm-level information and updating the particle motion based on prescribed rules.

To minimize an objective function (*f*) using PSO, each candidate solution is represented as a particle traveling in the solution space. At iteration *k*, the *i*th particle occupies a position, 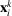, and exhibits a velocity, 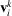. Each particle stores the best solution it has obtained thus far (referred to as its personal best, 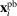). Via interparticle information sharing, the swarm also maintains the best solution obtained by any particle; this solution is referred to as the global best, **x**^gb^. The velocity of each particle is updated using equation (4), allowing stochastic convergence toward 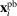 and **x**^gb^. Subsequently, the particle position is updated using the following equations, 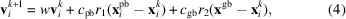

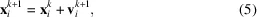
where *r*_1_ and *r*_2_ are independent and identically distributed random variables drawn from the uniform distribution, 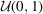. Parameter *w* represents a damping term that accounts for viscous resistance to particle motion. The coefficients *c*_pb_ and *c*_gb_ regulate the influences of 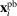 and **x**^gb^, respectively. Fig. 2 ▸ schematically illustrates the update of the particle position in PSO. In this figure, the circles represent particles, and the dashed arrows indicate the vectors involved in the velocity update described by equation (4).

### 3.2. Particle swarm optimization modifications

This subsection describes the modifications to the PSO algorithm to ensure rapid mirror alignment. The total alignment time is a sum of the mirror-movement and flux-measurement times. Since M1 and M2 are physically large, their motion is a dominant contributor to the total alignment time. To reduce the travel distance and number of mirror movements while maintaining search efficiency, we implement the following modifications:

(i) Particle initialization and dynamics.

(ii) Mirror-movement optimization.

(iii) Termination condition.

Details of each modification procedure are provided in the subsequent subsections.

#### 3.2.1. Particle initialization and dynamics

##### 3.2.1.1. Particle initialization

In standard PSO, particle positions are typically initialized randomly. Uneven initial distributions can introduce a bias in the search and increase the alignment time. To mitigate this effect, we initialize the particles via a combination of lattice-based and random placements. Specifically, when initializing *N* particles, we define *r* = 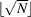. Thereafter, we place *r*^2^ particles on a regular lattice and place the remaining 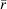 =*N* − *r*^2^ particles randomly. This approach reduces spatial bias by leveraging the *r*^2^ lattice points while maintaining stochasticity via the 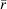 randomly placed particles.

##### 3.2.1.2. Particle dynamics

In PSO, particle motion is driven by synergistic attraction toward 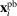 and **x**^gb^. However, during the middle stage of the search, particles may exhibit point-symmetric oscillations around **x**^gb^, which limits the exploration of a broader solution space. To suppress this behavior, we introduce a damping term that reduces particle velocity and introduce random noise to the velocity update. The damping coefficient is scheduled to be small and large during the early and late stages, respectively. This schedule enables rapid movement toward **x**^gb^ in the early stage, facilitating rapid particle convergence near **x**^gb^. In the late stage, stronger damping suppresses oscillations around **x**^gb^. Moreover, as particle velocities decrease, the relative contribution of the random noise increases, reducing the possibility of premature convergence to a local optimum in the late search stage.

##### 3.2.1.3. Measurement skip

Additionally, when a particle updates **x**^gb^, we bypass mirror motion and measurement for the remaining particles within that iteration, provided the newly obtained *f*(·) is at least 1.1 times larger than the previous **x**^gb^. While these remaining particles continue to update internally, their *f*(·) values are not evaluated on the beamline. This procedure minimizes the number of mirror motions. It is expected to shorten the overall alignment time while retaining particle-position updates.

#### 3.2.2. Mirror-movement optimization

During mirror-position alignment, the beam flux at each particle position must be measured on the beamline, and this requires the physical movements of the mirrors to the corresponding configurations. Because the time required for mirror motion is approximately proportional to the travel distance, a larger total travel distance will increase the alignment time. Fig. 3 ▸ illustrates the dependence of the travel distance on the measurement order.

Fig. 3 ▸(*a*) shows the mirror-motion trajectory in the parameter space when particles are evaluated based on their indices. Depending on the particle distribution, this ordering can produce a significant travel distance. Fig. 3 ▸(*b*) illustrates an example in which the particle evaluation order is rearranged to minimize the travel distance. In PSO, the order of evaluating particles within an iteration does not impact the optimization performance. By optimizing the particle-evaluation order to reduce the total mirror-travel distance, the time required for mirror-position alignment can be shortened without compromising the beam flux.

We propose a method that reorders the particle-evaluation sequence to minimize the total mirror-travel distance. Let the current mirror setting at iteration *k* be 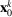, and let the mirror settings corresponding to *N* particles be 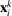 for *i* = 1,…, *N*. Determining a travel sequence that starts from 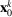 and visits the set, 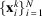, while minimizing the total mirror travel distance reduces the time required for mirror position alignment.

We formulate this sequence of mirror adjustments as a fixed-start open traveling salesman problem (TSP), a variant of the classical TSP (Dantzig *et al.*, 1954 ▸). Given a set of cities and the cost of traveling between them, the fixed-start open TSP seeks a minimum cost path that begins at a specified city, covers every city exactly once, and does not lead to the starting city. By treating each mirror setting as a city and defining a cost function *d*(·, ·) that represents the cost (distance) of moving between mirror settings, we compute a visiting order, σ, that minimizes the total distance as *D*(σ), 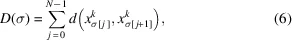
where σ is a list of indices corresponding to the mirror settings to be visited, with σ[0] = 0 because the sequence starts from the current mirror setting. Additionally, *d*(·, ·) is defined as the Manhattan distance on the two-dimensional plane of **x**. As M1 and M2 cannot be moved simultaneously in this study, the travel distance is defined as the sum of their individual travel distances. Notably, returning to the current mirror setting is not required in our problem setting; therefore, the path from the last setting, 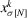, back to the current setting, 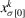, is not included in *D*(σ).

The open TSP is classified as a nondeterministic polynomial time-hard problem in the computational complexity theory. Consequently, obtaining an exact solution becomes challenging as the number of cities increases, corresponding to larger *N* in PSO. Therefore, we employ simulated annealing (Kirkpatrick *et al.*, 1983 ▸) to obtain an approximate solution.

#### 3.2.3. Termination condition

In PSO, the positions of all particles and their corresponding *f* are updated at each iteration. Consequently, even if the solution converges early, continuing the search for a predetermined number of iterations results in unnecessary mirror motions and flux measurements, which increase the alignment time. To mitigate this, we introduce an early stopping (ES) mechanism that terminates the search once convergence is detected. Under the ES mechanism, convergence is declared when **x**^gb^ remains unchanged for a specified number of consecutive iterations. Specifically, the search is terminated when **x**^gb^ has not been updated for *es* consecutive iterations and when the iteration count, *k*, exceeds a minimum number of iterations, *ei*. Parameter *ei* prevents premature termination during the early stages of the search, where further improvement is still possible. This mechanism suppresses redundant mirror motions and measurements after convergence, thereby reducing overall alignment time.

## 4. Simulation study

The proposed PSO-based algorithm was validated via simulation-based evaluations using a beam profile (a count map; Fig. 4 ▸) measured on the beamline. The beam profile was obtained by varying the mirror positions across a two-dimensional grid, followed by recording the resulting ionization-chamber-measured counts. The scan ranges were set to −3.49 mm ≤ *m*_1,*x*_ ≤ −0.79 mm and −1.15 mm ≤ *m*_2,*x*_ ≤ 1.55 mm, while the step size for both mirror motions was 0.1 mm. A total of 784 points were measured over 1 h.

The performance of the algorithm was evaluated using two metrics: beam flux and alignment time. Since the beam profile is known in this simulation, we assessed the beam flux based on the flux ratio, defined as the measured counts normalized by the maximum profile value. The alignment time is defined as the sum of the mirror travel time, the count-acquisition time, the command-execution time, and the algorithm-computation time. Based on preliminary timing measurements, the count-acquisition time was calculated using 1.0 s per measurement, whereas the command-execution time was calculated using 0.1–0.4 s per command depending on the command type. The mirror-travel time was approximately proportional to the total mirror-travel distance; accordingly it was estimated using a conversion factor of 6.5 s mm^−1^. The algorithm’s computation time was measured directly for each simulation trial. In this section, we evaluate the effects of the proposed modifications to PSO and compare the results with a general-purpose GP–BO implementation to gain insight into the characteristics underlying the effectiveness of the proposed PSO method.

### 4.1. Ablation study

This subsection presents an ablation study designed to quantify the contribution of each modification to the performance of the proposed method. The termination condition (TC), described in Section 3.2.3, is known to induce a trade-off between alignment time and the realized beam flux. Accordingly, three configurations are compared for this study:

(i) **Baseline**: PSO without any modifications.

(ii) **without termination condition (woTC)**: particle initialization and dynamics (Section 3.2.1) + mirror-move optimization (Section 3.2.2).

(iii) **Proposed**: all improvements described in Section 3.2.

For each method and swarm size, *N*, 1000 independent simulations were run. The resulting alignment times and flux ratios were compared across the various conditions. Fig. 5 ▸ presents the results for swarm sizes *N* = (5, 10, 15 and 20).

Based on preliminary tests, the early stopping parameters shown in Section 3.2.3 were set to *es* = 3 and *ei* = 5, simultaneously satisfying practical time constraints and maintaining an adequate beam flux.

The ES mechanism caused the alignment time to vary across trials. The alignment time and post-alignment beam flux were evaluated separately. In the simulations, the maximum attainable beam flux was known; therefore, the final flux ratio provided a direct measure of how closely the alignment approached this maximum. The final flux-ratio distribution is presented as the proportion of trials falling within predefined bins, whereas the alignment time is presented in Appendix A3[App appa].

Fig. 5 ▸(*a*) presents a stacked bar chart of the post-alignment beam flux. The horizontal axis denotes the employed optimization method, and the vertical axis represents the fraction of trials assigned to predefined flux-ratio bins. The colors correspond to flux-ratio bins indicated in the legend. Higher and lower bar segments correspond to lower and higher flux-ratio bins, respectively. A larger fraction of trials in bins near 1.0 indicates that more trials achieved the maximum flux of the profile.

A comparison of **Baseline** with **woTC** reveals that the fraction of trials attaining the maximum flux increased across all *N* values, demonstrating that the proposed modifications enhanced the ability to locate the profile maximum. Conversely, a comparison of **woTC** with **Proposed** reveals a decrease in the fraction of trials reaching the maximum flux, indicating that **woTC** introduces a trade-off between alignment time and the achieved beam flux.

Fig. 5 ▸(*b*) compares the alignment-time distributions across methods. The plot is presented as a box-and-whisker diagram, where the box spans the first to the third quartiles, with the line inside it indicating the median. The whiskers extend to the minimum and maximum values within 1.5 times the interquartile range of the box edges; values beyond this range are shown as outliers.

Overall, for all values of *N*, the median alignment time followed the order **Baseline** > **woTC** > **Proposed**. At *N* = 15, the median alignment time decreased from 744.1 s for **woTC** to 319.2 s for **Proposed**, corresponding to a median reduction of 424.9 s (95% bootstrap confidence interval: 415.5–433.4 s). The median alignment time for **Proposed** remained close to the 5 min target.

Although **Proposed** with *N* = 20 achieved the highest proportion of trials with a flux ratio of >0.99 (87.2%; 95% exact binomial confidence interval: 85.0–89.2%), its median alignment time exceeded the target of 5 min. Meanwhile, **Proposed** with *N* = 15 maintained a high proportion of near-maximum-flux trials (77.0%; 95% exact binomial confidence interval: 74.3–79.6%), while keeping the median alignment time close to the target. Therefore, we selected *N* = 15, together with *es* = 3 and *ei* = 5, as a practical operating point that balances beam flux and alignment time rather than optimizing either metric alone.

### 4.2. Comparison of PSO with BO

This subsection compares the proposed PSO method with a standard, general-purpose GP–BO implementation. GP–BO was selected because it searches for the optimum using a surrogate model and an acquisition function, representing a search strategy that differs fundamentally from the population-based collective search performed by PSO. This comparison provides insight into searching characteristics that contribute to the effectiveness of the proposed method for mirror alignment. The following sections describe the evaluated GP–BO implementation, the optimization conditions and evaluation metrics, and present a comparative analysis of the simulation results.

#### 4.2.1. Bayesian optimization method

BO is a black box optimization methodology for objective functions exhibiting an unknown internal structure, similar to PSO. This methodology constructs a surrogate model that estimates the objective function (in this case, the beam-flux distribution over the search space), using evaluation results obtained during the optimization. Consequently, the surrogate model probabilistically guides the selection of the subsequent evaluation point, enabling efficient convergence without exhaustive search-space sampling. Therefore, BO is particularly effective for problems involving cost- and time-intensive evaluations, as is often the case in experiments and numerical analyses.

In this study, we implemented the mirror-alignment algorithm using scikit-optimize (Head *et al.*, 2024 ▸), a widely adopted Python library for BO. We implemented a Gaussian process as the surrogate model, and the gp_hedge acquisition function was applied to adaptively balance exploration and exploitation.

#### 4.2.2. Simulation setup

Similar to the previous subsection, the evaluation metrics included the flux ratio and alignment time. Each case comprised 100 independent simulation trials.

For GP–BO, four configurations were evaluated to analyze the effects of surrogate-model fidelity and search effort (Table 1 ▸). These configurations combined the number of initial evaluations (10 or 30) with the ES patience (10 or 20). A higher number of initial evaluations enhances the accuracy of the surrogate, although it increases the initialization time. Conversely, greater patience allows a more extensive search, potentially improving convergence, although it increases alignment times. The evaluated configurations differed only in these two settings; no exhaustive selection or optimization of the Gaussian-process model, kernel, acquisition function, or their associated hyperparameters was performed.

For PSO, we utilized *N* = 15, *es* = 3, and *ei* = 5, which were identified as practical configurations in Section 4.1.

#### 4.2.3. Simulation results

Fig. 6 ▸ presents a comparison between the proposed PSO and the evaluated GP–BO configurations. The bar chart [Fig. 6 ▸(*a*)] shows a comparison of the numbers of runs grouped by flux-ratio bins, with each color denoting a specific flux-ratio range. The box-and-whisker plots in Fig. 6 ▸(*b*) illustrate the comparison of the alignment-time distributions of PSO and GP–BO.

Fig. 6 ▸(*a*) shows that PSO consistently achieved high flux ratios across all runs. Conversely, the evaluated GP–BO configurations exhibited a trade-off between the attained flux ratio and alignment time. Additionally, across the evaluated GP–BO configurations, the attained flux ratios exhibited substantial variability, with some runs converging to low-flux solutions (flux ratio < 0.5).

The objective landscape was characterized by a broad, low-flux plateau and a narrow, anisotropic high-flux peak (Fig. 4 ▸). Because the peak occupies only a small fraction of the search space, the evaluated GP–BO configurations likely sampled observations predominantly from the flat region in some runs, providing limited information to the Gaussian-process surrogate and thereby contributing to the observed variability in the attained flux ratios. Meanwhile, PSO distributed multiple particles throughout the search space and iteratively updated them toward the global-best position. This search strategy increased the likelihood of one or more particles encountering the narrow high-flux region; once such a point was identified, the swarm concentrated its search around that region. This behavior plausibly explains the robust performance of PSO observed in the simulations.

To examine how the distinct evaluation patterns of PSO and GP–BO configurations influenced alignment time, the mean alignment-time breakdown for each method was determined (Fig. 7 ▸). The count-acquisition, command-execution, mirror-travel, and algorithm-computation times were averaged over 100 independent simulation trials.

Mirror travel accounted for ∼83–85% of the mean alignment time in the evaluated GP–BO configurations, compared with ∼33% for PSO. Moreover, count acquisition and command execution accounted for ∼35% and 32% of the total alignment time in PSO, respectively, indicating that these three components contributed comparably to the overall alignment time. The mean mirror-travel time was ∼135 s for PSO, whereas it ranged from 286 to 666 s for GP–BO. Algorithm computation accounted for <2% of the alignment time in all cases. Although the shorter count-acquisition times indicate that the evaluated GP–BO configurations required fewer objective-function evaluations than PSO, physical mirror movement between successive evaluation points remained the primary contributor to their alignment time.

Because mirror-travel time was estimated from the total travel distance, the differences between the two approaches reflected their distinct motion trajectories. The GP–BO implementation selected points without accounting for movement cost relative to the current mirror setting, allowing successive points to be widely separated. Meanwhile, PSO generated particle positions for each iteration in advance and optimized the order of their evaluations. Combined with the swarm’s inherently convergent search behavior, this evaluation-order optimization reduced the total travel distance and consequently the mirror-travel time.

The scope of this comparison is limited to the standard, general-purpose GP–BO implementation evaluated under the tested simulation conditions. Accordingly, the results characterize differences in search behavior between the proposed PSO method and this GP–BO baseline for the mirror-alignment problem rather than establishing the general superiority of PSO over BO. More specialized BO approaches incorporating beamline-specific coordinate transformations, latent parameterizations, custom kernels, physical or probabilistic constraints, transfer learning, prior operational data, or failure-aware acquisition strategies are beyond the scope of this study and may exhibit different performance.

## 5. Beamline implementation and operational results

This section describes the implementation of the proposed method on beamlines and performance evaluation during routine operation. The optimization algorithm was implemented in Python as a hardware-independent module, decoupled from the beamline control layer and GUI. Mirror motion and beam-flux acquisition were performed in LabVIEW and were exposed to the optimizer algorithm through a dedicated Python library that abstracted these facility-specific operations. The GUI was also implemented independently in LabVIEW. Consequently, deploying the system at another facility requires only adapting the interface to the local control system while the core optimization algorithm can be reused without fundamental modification.

Fig. 8 ▸ presents a screenshot of the developed GUI, which executes start, pause, resume, and abort operations with a single click. The lower panel displays the alignment iteration number and the current count value. The GUI also executes an automatic rollback function that restores the pre-alignment state when the run is aborted or when the alignment causes a decrease in beam flux. Consequently, utilizing this tool does not exert any adverse side effects.

For the evaluation, execution logs of the GUI tool were collected over 15 months (14 April 2024 to 15 July 2025). The beam-flux ratio (after/before alignment) and the time required to complete the alignment were analyzed. During this period, the GUI tool was executed 124 times. The tool was primarily used in the following situations:

(i) When optical alignment was required, for example after switching the monochromator crystal or changing the mirror angle.

(ii) When the beamline had been operated for an extended period under stable optical conditions, such as after long measurements.

(iii) When an evident decrease in beam flux was observed.

Fig. 9 ▸(*a*) presents a histogram of beam-flux ratios. Out of 124 runs, beam flux remained unchanged in 17 cases (13.7%), whereas an increase was achieved in 107 cases (86.3%). These results indicate that the proposed method generally enhances beam flux. Notably, the rollback function prevented any beam-flux reduction, even in the 17 runs that showed no improvement. The 17 cases comprised reruns under already adjusted conditions and additional runs conducted for operator training.

Fig. 9 ▸(*b*) presents a histogram of the alignment times. The modal interval was 4–6 min, involving 48 runs (38.7%). Further analysis of the execution logs revealed that the alignment time depended on the mirror-travel distance for each run. Notably, users did not abort runs even when the alignment time exceeded 5 min, indicating that durations longer than 5 min may still be acceptable.

## 6. Discussion and conclusions

An automated optical-alignment algorithm and GUI tool were developed for slow mirror systems to support routine operation at the SPring-8 Toyota beamline, BL33XU, compensating for decreases in synchrotron X-ray flux caused by variations in storage-ring operating conditions. The objective function was defined based on an operational requirement: simultaneous control of two mirrors to restore the maximum-flux beam within a 5 min window. The alignment algorithm is based on particle swarm optimization (PSO). Algorithmic modifications were introduced to reduce mirror movement and the number of search-and-evaluation steps, thereby shortening alignment time while maximizing beam flux. In the simulations, these modifications shortened the alignment time relative to the baseline PSO configuration while maintaining high beam flux. The proposed method also produced more consistent beam-flux ratios and shorter alignment times than a GP–BO implementation. However, this comparison was limited to the evaluated configurations.

Furthermore, the proposed method was validated through continuous operation of an automated mirror-position-alignment system at the beamline, enabling the rapid restoration of a high-flux beam. During a 15 month deployment on an operational synchrotron beamline (124 executions), beam flux improved in 107 runs (86.3%), demonstrating the operational robustness and reliability of the proposed alignment method.

Future studies will extend the proposed method to other optical elements and more complex alignment problems. Although the PSO formulation can accommodate additional variables, higher-dimensional or strongly multimodal optimization landscapes become increasingly challenging to search because the swarm converges toward the current global-best position. One promising approach is to decompose the alignment task into lower-dimensional subproblems and apply optimization methods best suited to each task. In addition, measurement noise may reduce the reliability of beam-flux evaluations and beam drift may shift the optimum during an optimization run. Rapid, low-overhead realignment can mitigate the effect of such drift. These extensions, including the simultaneous and multi-objective optimization of multiple optical elements, would further broaden the practical applicability of the proposed method.

## Figures and Tables

**Figure 1:**
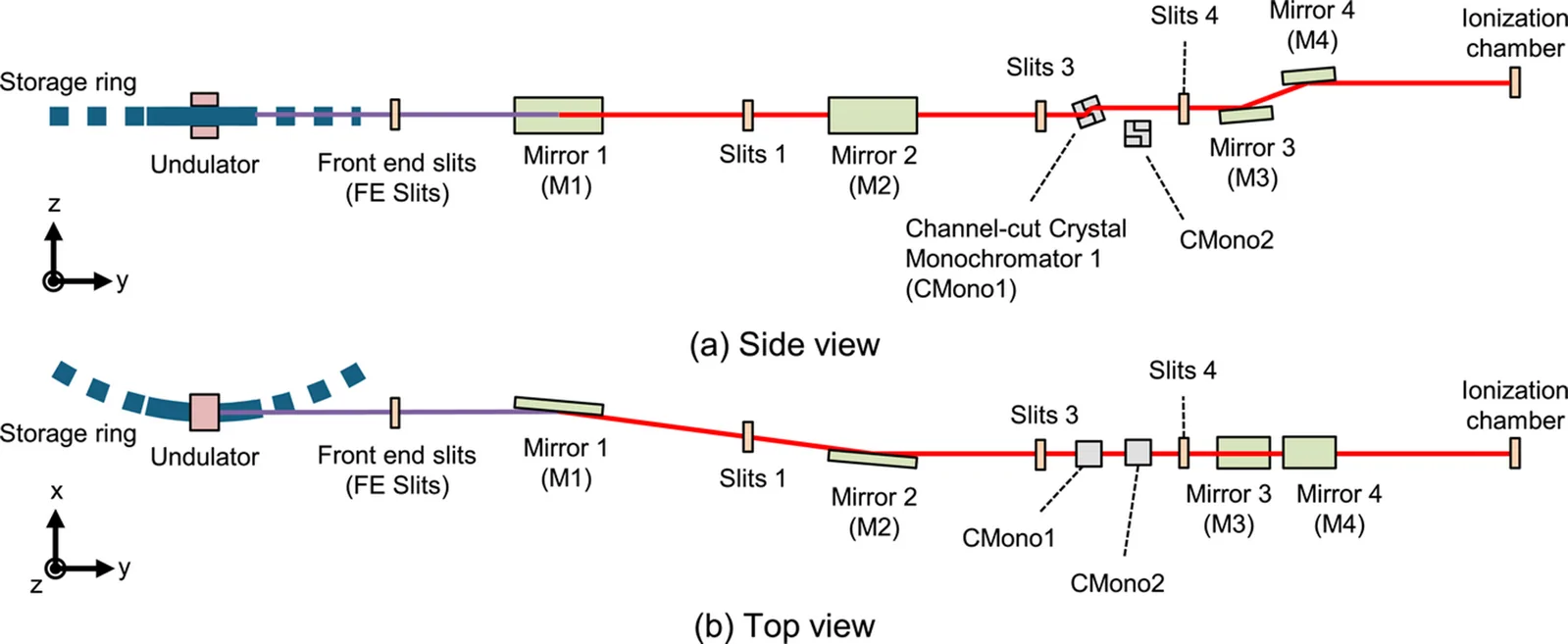
Optics 1 in the Toyota beamline (BL33XU). The beam is delivered to the sample by multiple mirrors and slits. The synchrotron X-ray flux is measured by an ionization chamber installed at the sample position.

**Figure 2:**
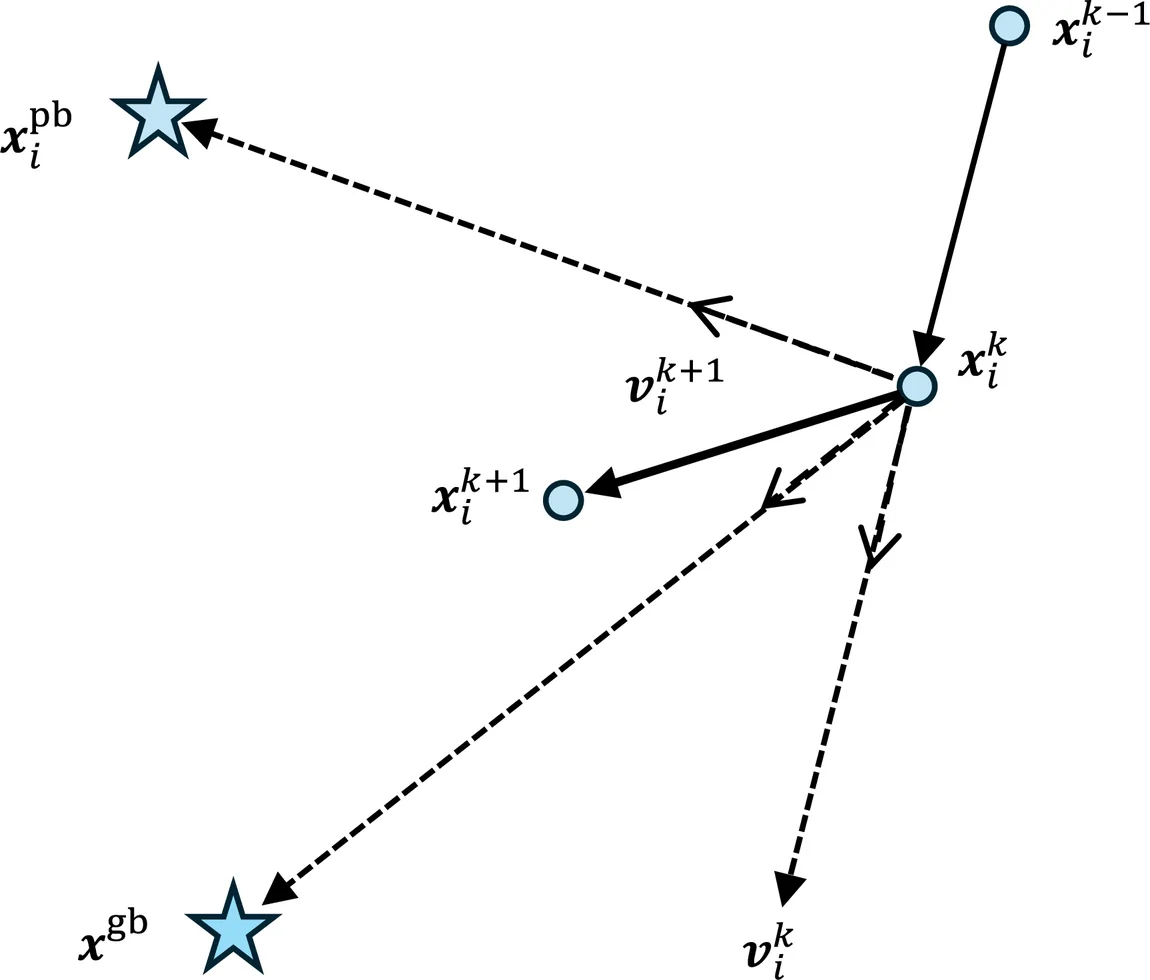
Schematic of particle-position update. We use *w* = 0.8 with decay, *c*_pb_ = 0.5, *c*_gb_ = 0.5.

**Figure 3:**
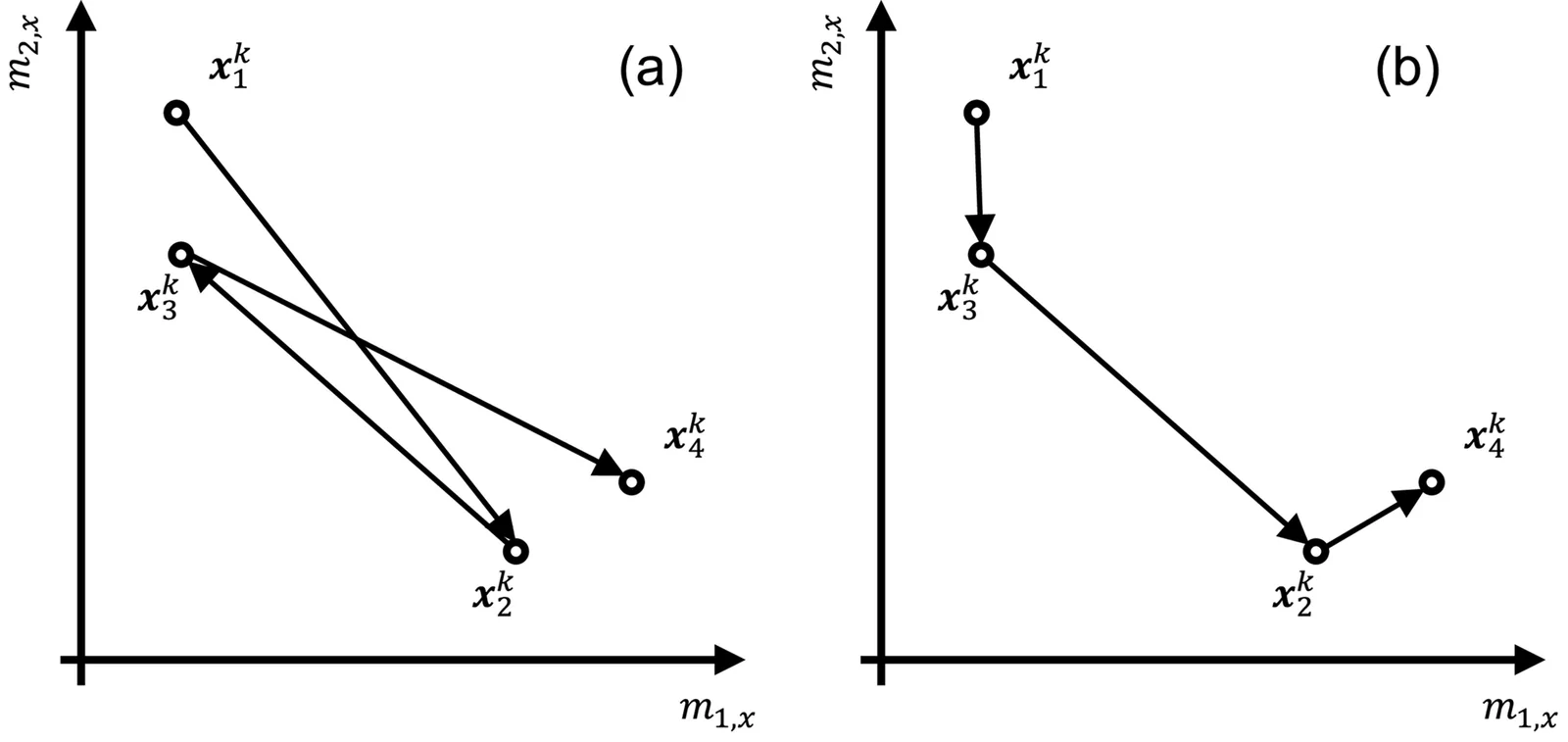
Impact of particle-evaluation order in the particle swarm optimization (PSO) framework on the total mirror-movement distance.

**Figure 4:**
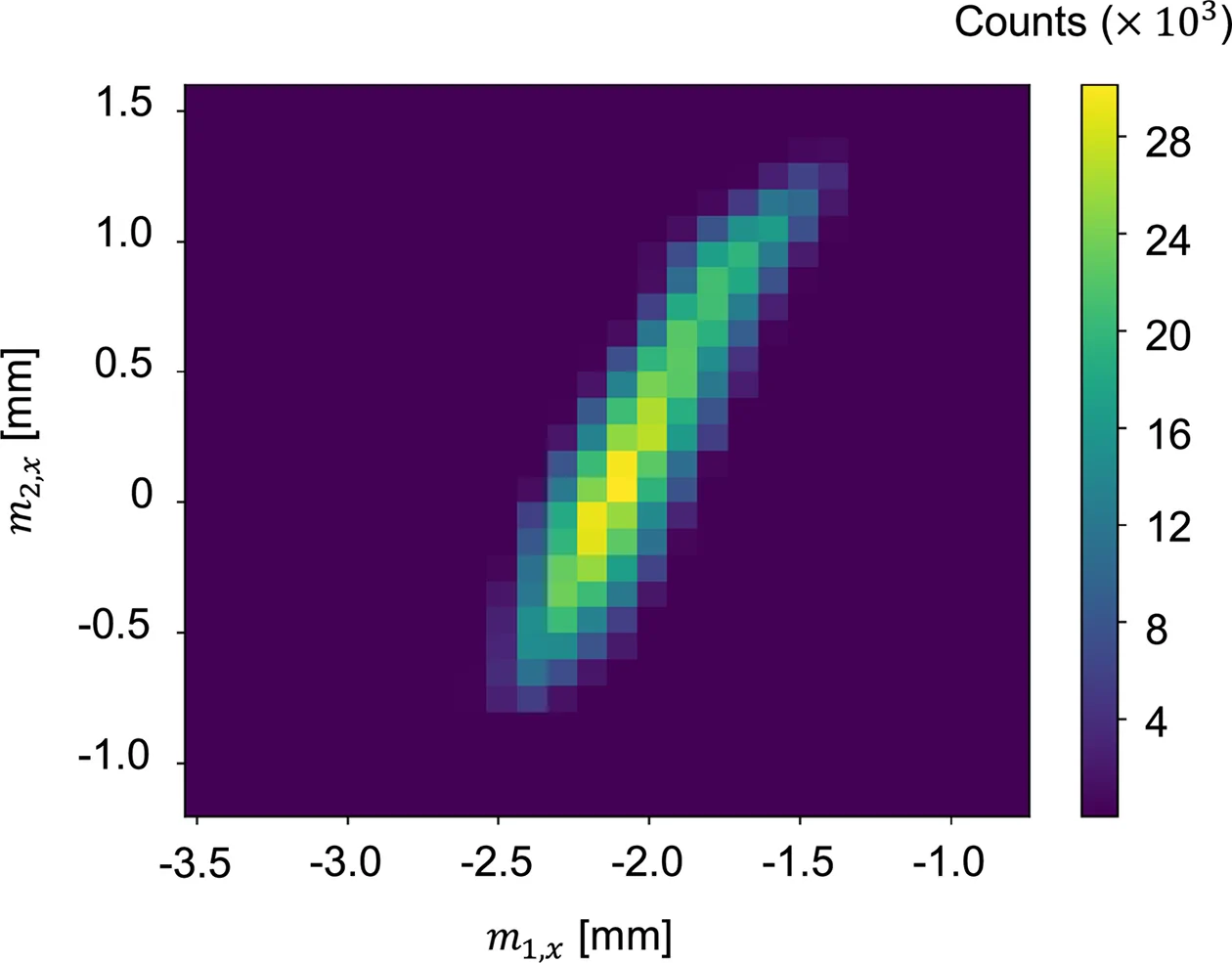
Beam profile for simulation obtained by varying the mirror positions across a two-dimensional grid. A total of 784 points were measured over 1 h with the step size of 0.1 mm.

**Figure 5:**
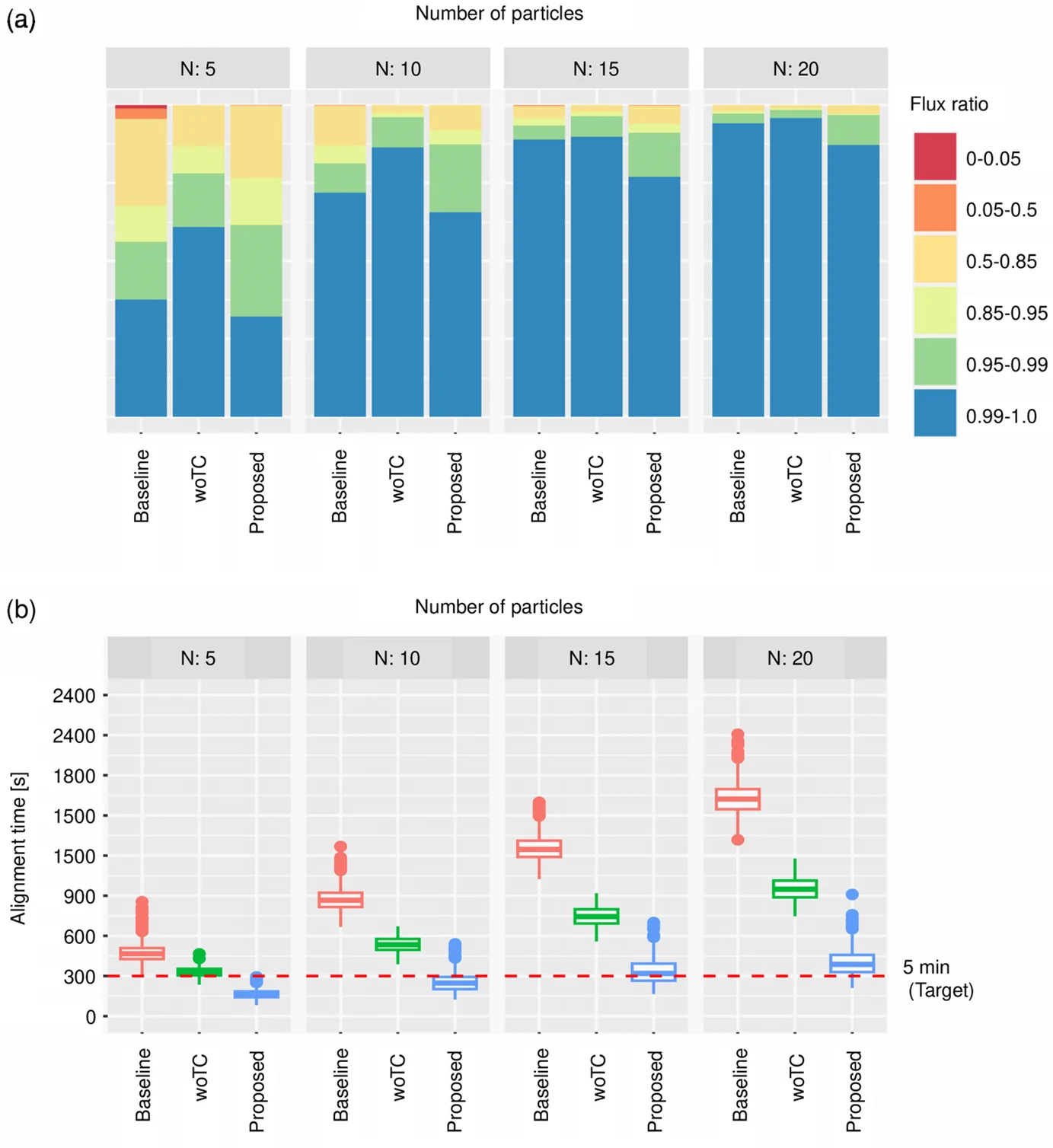
Comparison of the alignment times and beam-flux ratios across different *N* values in **Baseline**, **woTC** and **Proposed**. Panel (*a*) compares the distributions of beam-flux ratios, whereas panel (*b*) compares the distributions of alignment times. The red dashed line in (*b*) indicates the target alignment time of 5 min.

**Figure 6:**
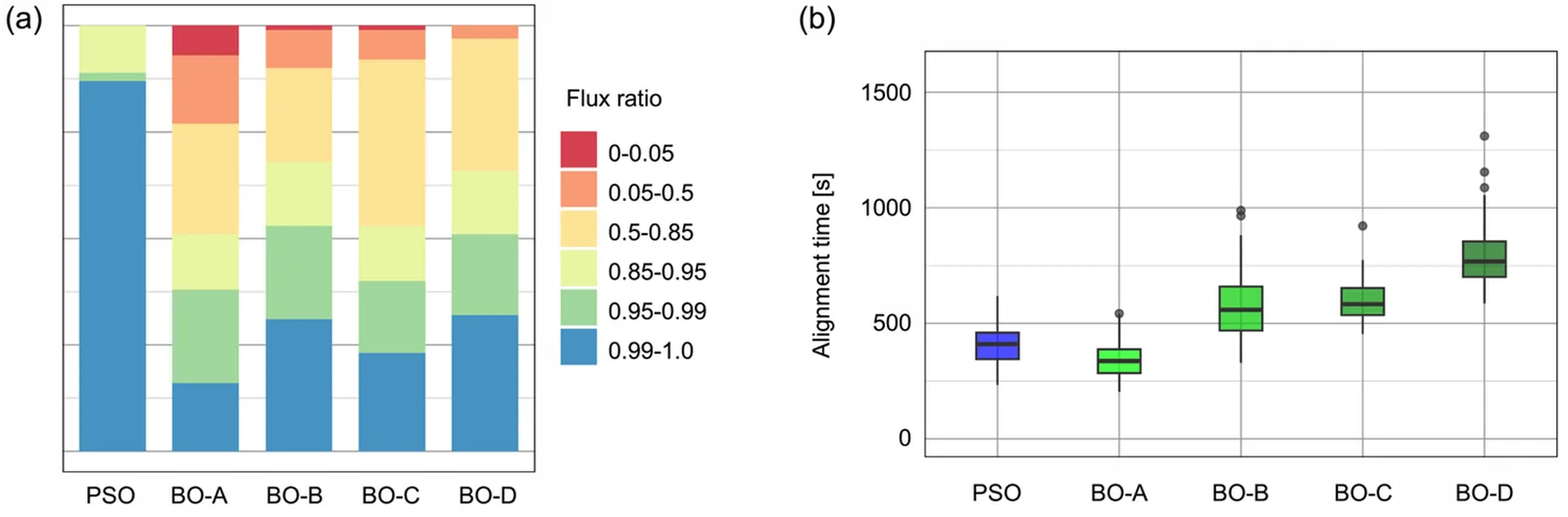
Comparison of PSO and the evaluated GP–BO configurations. (*a*) Numbers of runs grouped by attained flux-ratio bins. (*b*) Distributions of alignment times. Each case comprises 100 independent simulation trials.

**Figure 7:**
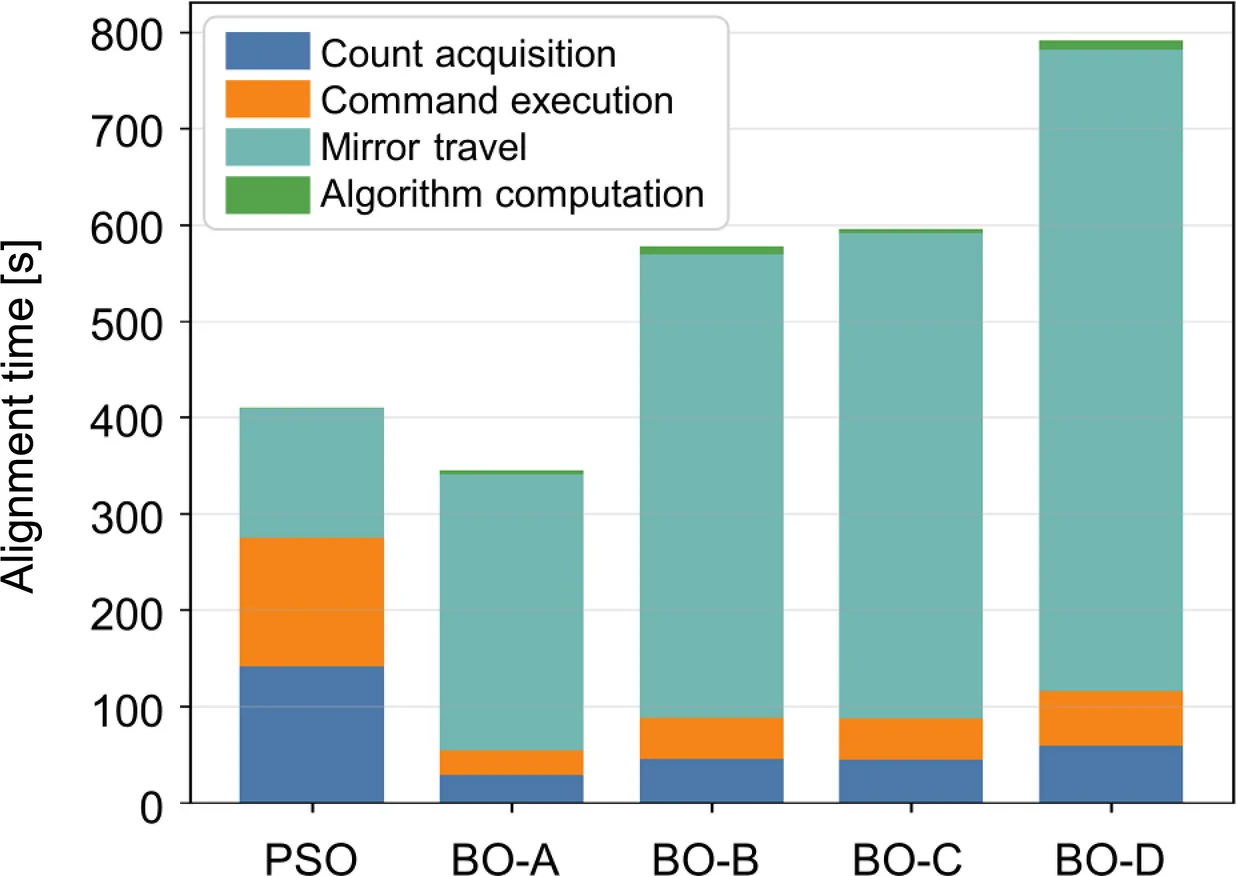
Breakdown of the estimated mean alignment time for PSO and each evaluated GP–BO configuration. Each stacked bar represents the mean duration of each time component over 100 independent simulation trials.

**Figure 8:**
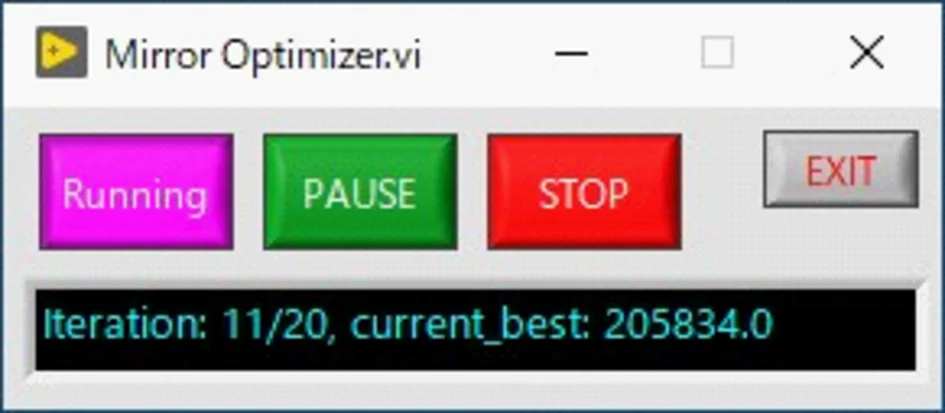
Graphical user interface (GUI) for mirror optimization. The GUI provides one-click control, displays the alignment iteration number and current count value, and includes automatic rollback to the pre-alignment state when the run is aborted or beam flux decreases.

**Figure 9:**
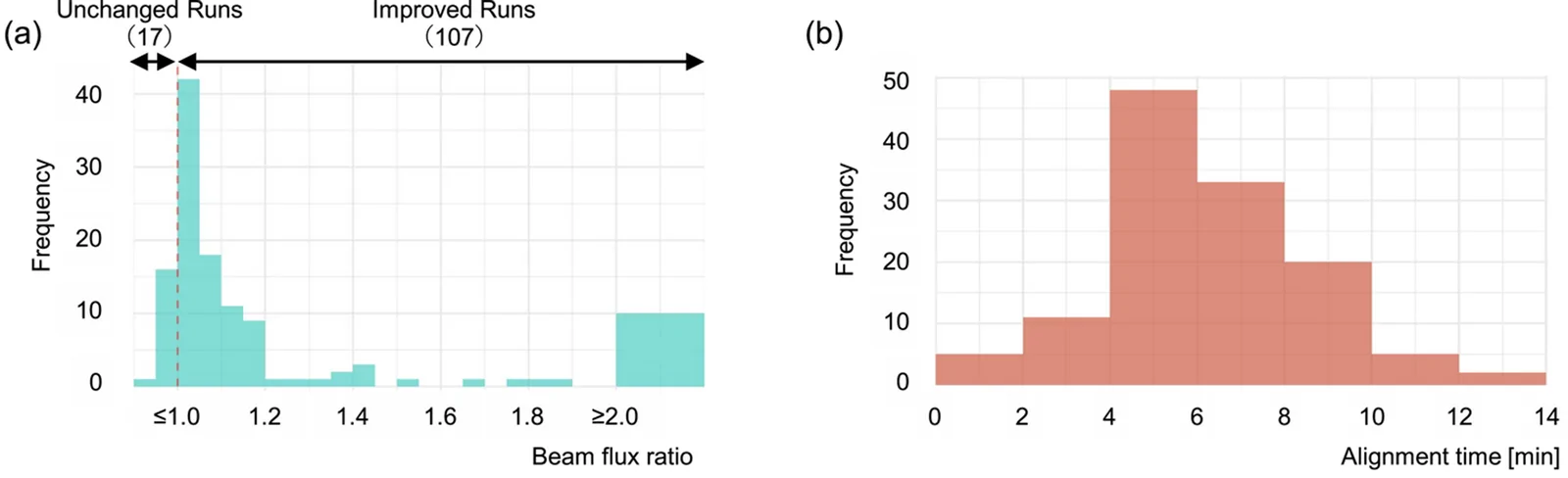
Operational results of the GUI tool based on 124 execution logs collected over 15 months (14 April 2024 to 15 July 2025): (*a*) frequency distribution of the beam-flux ratios, with 107 runs with improvements in the beam-flux ratio and 17 runs with no changes in the parameter; (*b*) frequency distribution of the alignment times, with a modal interval of 4–6 min.

**Figure 10:**
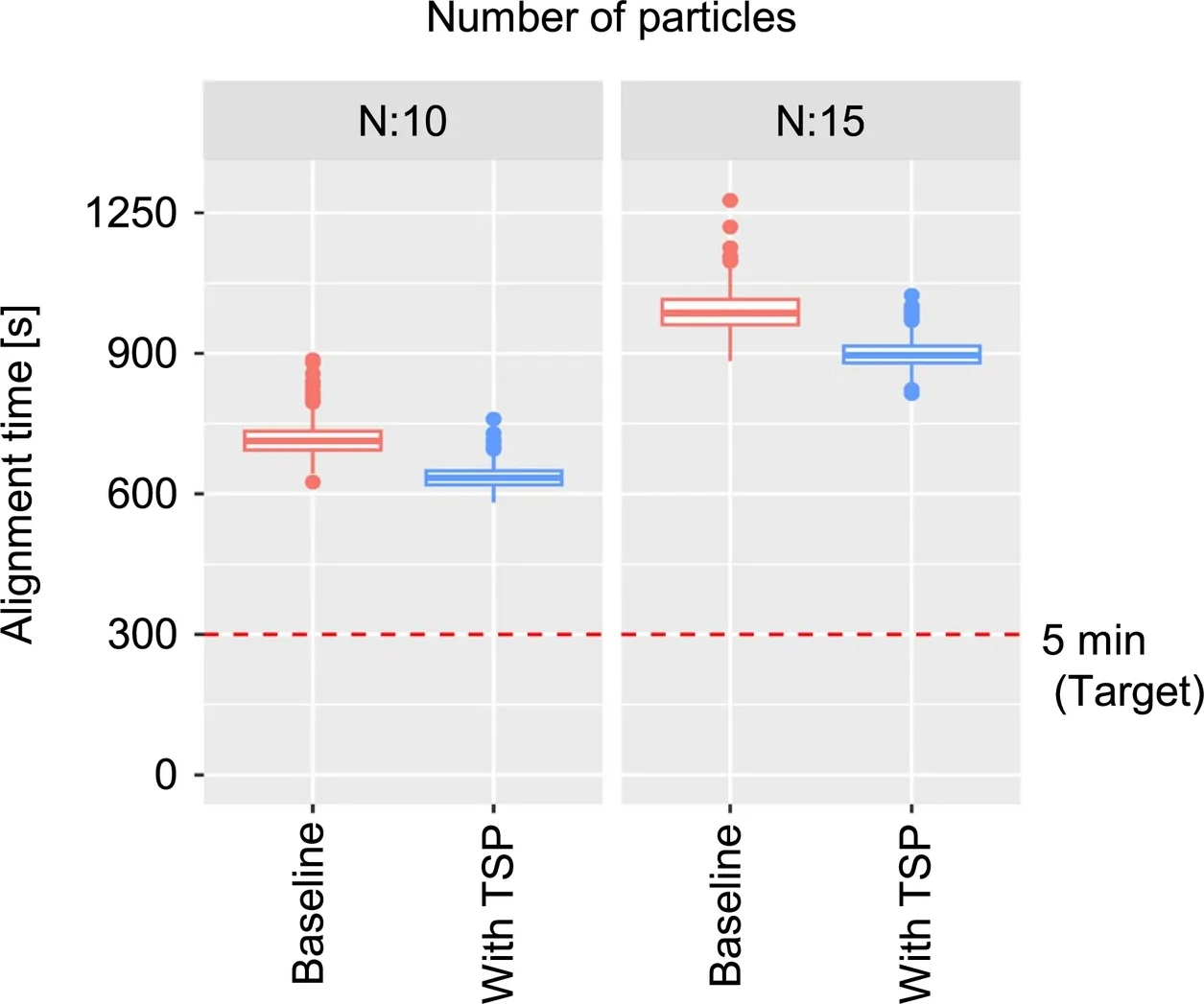
Effect of mirror-movement optimization on mirror-position-alignment time for (*a*) *N* = 10 and (*b*) *N* = 15. Each panel compares the **Baseline** method with the method incorporating traveling-salesman-problem-based mirror-movement optimization (**With TSP**). The red dashed line indicates the target alignment time of 5 min.

**Figure 11:**
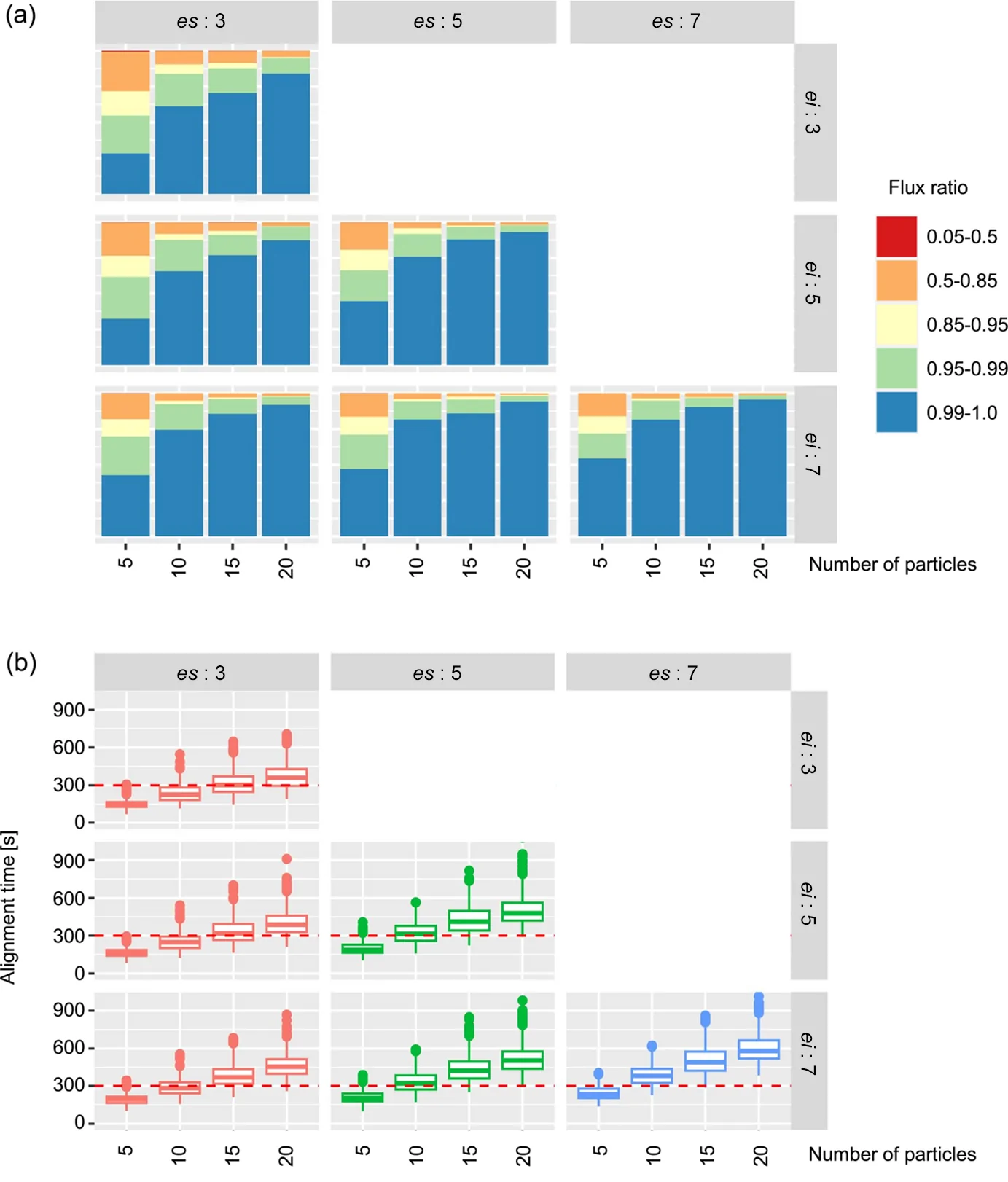
Comparison of alignment time and flux ratio as functions of the ES parameters (*es* and *ei*) and *N*. (*a*) Distribution of the beam-flux ratio relative to the maximum beam flux. (*b*) Distribution of the alignment time.

**Figure 12:**
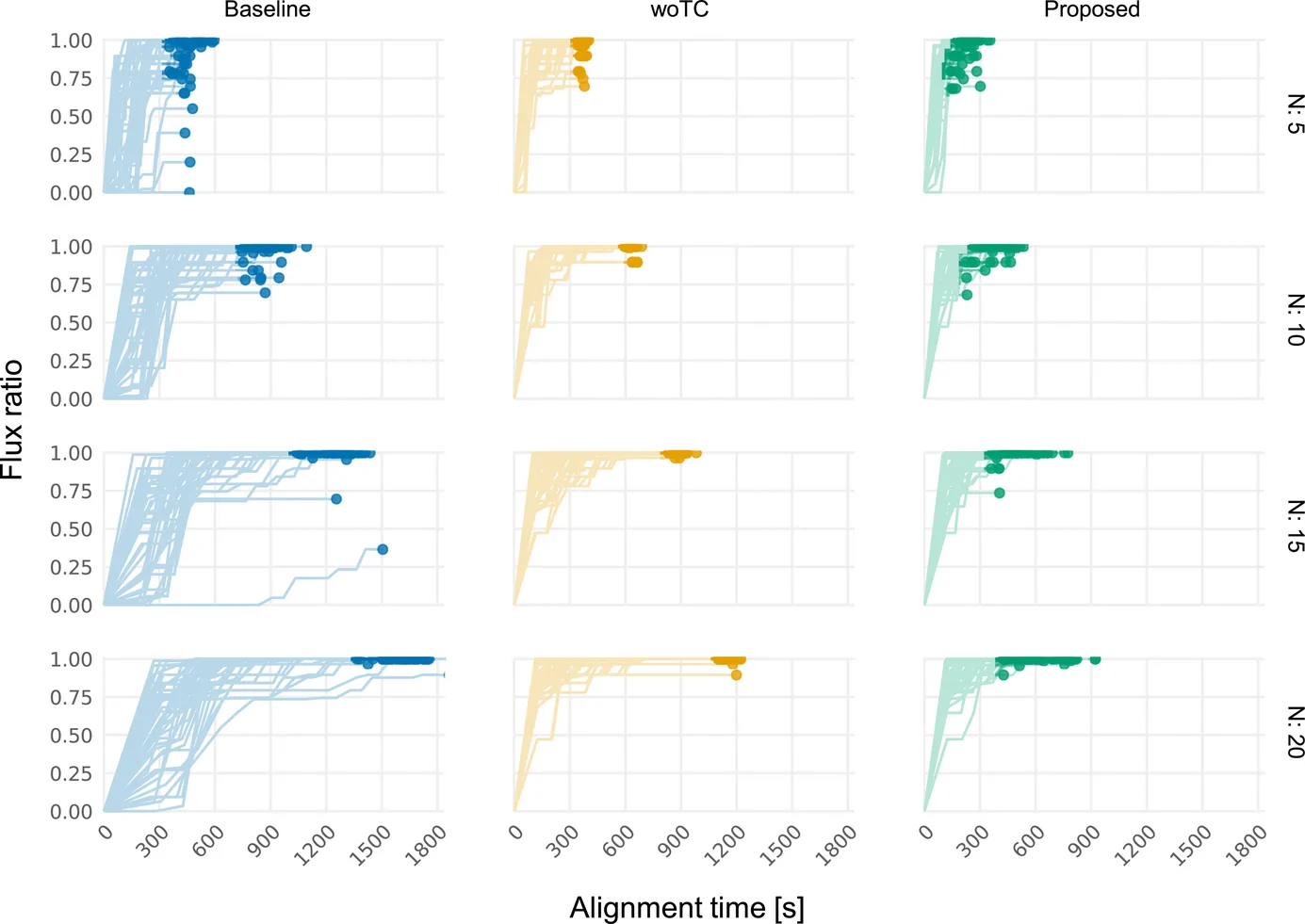
Convergence trajectories of the flux ratio as a function of the alignment time for the **Baseline**, **woTC**, and **Proposed** configurations. The columns correspond to the three configurations, and the rows correspond to swarm sizes *N* = 5, 10, 15, and 20. Each curve represents one of 50 randomly selected simulation trials (out of the 1000 trials reported in Section 4.1), and the marker indicates the termination point.

**Table 1:** Parameter configurations for the evaluated GP–BO implementation

Case	Initial evaluations	Early stop patience
A	10	10
B	10	20
C	30	10
D	30	20

## Data Availability

The datasets during the study are available from the corresponding author upon reasonable request.

## Appendix A Additional simulation results

### A1. Effect of mirror-movement reduction on the alignment time

In this subsection, we evaluate the effect of the mirror-movement optimization described in Section 3.2.2. Since this modification does not impact beam-flux performance, we employ the time for aligning the mirror position as the evaluation metric. This metric is defined as the total alignment time and includes the computation time required to solve the open TSP.

Fig. 10 ▸ compares the mirror-position-alignment times for *N* = 10 and 15. The red dashed line indicates the target alignment time of 5 min. The figure shows that mirror-movement optimization shortens the alignment time for both *N* values.

### A2. Performance evaluation using reduced mirror movement and evaluation counts

Reductions in mirror movement and the number of evaluations are incorporated into the PSO algorithm. In this subsection, performance is evaluated as a function of the following hyperparameters: the ES threshold, *es* (where the search terminates when the objective function remains unchanged for *es* consecutive iterations); the minimum number of iterations before enabling ES, *ei*; and the number of particles.

For each parameter combination, 1000 simulations were conducted, and the results are summarized in Fig. 11 ▸. Fig. 11 ▸(*a*) presents the distribution of the beam-flux ratio relative to the maximum beam flux, whereas Fig. 11 ▸(*b*) illustrates the alignment-time distribution. Each panel corresponds to a different (*es*, *ei*) configuration, where *es*, *ei* ∈ {3, 5, 7}, and presents results for particle numbers *N* ∈ {5, 10, 15, 20}. For each configuration, ES is activated at iteration *ei*, and the search is terminated when the maximum beam flux is not updated for *es* consecutive iterations. Within these configurations, the effects of *N* on the alignment time and beam-flux ratio were compared.

#### A2.1. Effect of *N*

Fig. 11 ▸(*a*) demonstrates that higher *N* yields a higher beam-flux ratio. As illustrated in Fig. 11 ▸(*b*), the alignment time increases with the increasing *N*. These results indicate a trade-off between alignment time and beam flux across the considered *N* range.

#### A2.2. Effects of search-iteration parameters *es* and *ei*

Fig. 11 ▸(*a*) demonstrates that larger *es* and *ei* values tend to increase the beam-flux ratio. Fig. 11 ▸(*b*) reveals that smaller *es* and *ei* tend to shorten the alignment time, as ES is applied more aggressively. These findings highlight a trade-off between the alignment time and beam flux across the considered *es* and *ei* ranges.

As shown in Fig. 11 ▸, when *es* = 3 with *ei* = 3 or *ei* = 5, the median alignment time exceeds 5 min, the operational target, at the maximum *N* (20). To prevent extremely aggressive early termination at the initial search stage, we adopt *ei* = 5 in this study. Under this configuration, the distribution of the beam-flux ratio does not deviate substantially from that observed across other (*es*, *ei*) configurations. Therefore, *es* = 3 and *ei* = 5 are considered effective ES conditions that meet the time constraint for practical operation while maintaining the beam flux.

### A3. Convergence behavior

Fig. 12 ▸ shows the flux-ratio trajectories obtained from individual simulation trials. Each curve represents a single trial, and its endpoint indicates the flux ratio and alignment time at termination. Thus, the figure illustrates the convergence process and trial-to-trial variability at the termination moment.

For the **Baseline** method, increasing the swarm size *N* not only generally improved the final flux ratio but also increased the alignment time, indicating a trade-off between the attainable flux ratio and alignment time. The **woTC** method substantially mitigated this trade-off. Across the evaluated swarm sizes, **woTC** exhibited a more rapid increase in the flux ratio and achieved values closer to 1.0 in shorter alignment times than **Baseline**. **Proposed** further reduced the alignment time by terminating trials when the prescribed ES criteria were met. As shown in the trajectories, alignment was completed within ∼300 s for *N* = 5 and 900 s for *N* = 15 while maintaining final flux ratios comparable with those achieved by **woTC**.
